# Expanding Global Surgery Education in Brazil: Perspectives after the 35^th^ Brazilian Surgical Congress

**DOI:** 10.1590/0100-6991e-20243667-en

**Published:** 2024-01-10

**Authors:** LUIZA TELLES DE ANDRADE ALVARES, AYLA GERK RANGEL, LETÍCIA NUNES CAMPOS, SOFIA WAGEMAKER VIANA, ANA WOO SOOK KIM, NATÁLIA ZANETI SAMPAIO, ROSEANNE FERREIRA, JOAQUIM BUSTORFF SILVA, DAVID P MOONEY, CRISTINA PIRES CAMARGO

**Affiliations:** 1 - Instituto de Educação Médica (IDOMED), Campus Vista Carioca - Rio de Janeiro - RJ - Brasil; 2 - Harvard Medical School, Program in Global Surgery and Social Change - Boston - Massachusetts - Estados Unidos; 3 - Universidade Federal de Pernambuco - Recife - PE - Brasil; 4 - Kursk State Medical University - Kursk - Kurskaya Oblast - Rússia; 5 - Centro Universitário São Camilo - São Paulo - SP - Brasil; 6 - Universidade de Araraquara - Araraquara - SP - Brasil; 7 - McMaster University, Department of Health Research Methods, Evidence and, Impact - Hamilton - Ontario - Canadá; 8 - Universidade Estadual de Campinas (UNICAMP) - Campinas - SP - Brasil; 9 - Boston Children’s Hospital - Boston - Massachusetts - Estados Unidos; 10 - Universidade de São Paulo, Faculdade de Medicina - São Paulo - SP - Brasil

**Keywords:** Global Burden of Disease, Global Health Strategies, Health Equity, Quality Indicators, Health Care, Health Workforce, Cirurgia Geral, Acesso aos Serviços de Saúde, Educação, Indicadores de Qualidade em Assistência à Saúde

## Abstract

The 35^th^ Brazilian Congress of Surgery marked a turning point for surgical education in the country. For the first time, the Brazilian College of Surgeons included Global Surgery on the main congressional agenda, providing a unique opportunity to rethink how surgical skills are taught from a public health perspective. This discussion prompts us to consider why and how Global Surgery education should be expanded in Brazil. Although Brazilian researchers and institutions have contributed to the fields expansion since 2015, Global Surgery education initiatives are still incipient in our country. Relying on successful strategies can be a starting point to promote the area among national surgical practitioners. In this editorial, we discuss potential strategies to expand Global Surgery education opportunities and propose a series of recommendations at the national level.

## RESUMO

O 35º Congresso Brasileiro de Cirurgia foi marcado por discussões inovadoras para a educação cirúrgica no país. Pela primeira vez, o Colégio Brasileiro de Cirurgiões incluiu a Cirurgia Global na pauta principal do congresso, proporcionando uma oportunidade única de repensar como as habilidades cirúrgicas são ensinadas a partir de uma perspectiva de saúde pública. Essa discussão nos leva a considerar por que e como o ensino da Cirurgia Global deve ser expandido no Brasil. Embora pesquisadores e instituições brasileiras tenham contribuído para a expansão do campo desde 2015, as iniciativas de educação em Cirurgia Global ainda são incipientes em nosso país. Basear-se em estratégias bem-sucedidas pode ser um ponto de partida para promover a área entre os profissionais de cirurgia nacionais. Neste editorial, discutimos potenciais estratégias para expandir as oportunidades de educação em Cirurgia Global e propomos uma série de recomendações a nível nacional.

The 35^th^ Brazilian Congress of Surgery marked a turning point for surgical education in the country. For the first time, the Brazilian College of Surgeons included Global Surgery on the main congressional agenda, providing a unique opportunity to rethink how surgical skills are taught from a public health perspective. Throughout the event, there was a consistent focus on how Global Surgery programs can improve surgical accessibility, emphasizing the urgent need to broaden the field and address pressing health disparities. This discussion prompts us to consider why and how Global Surgery education should be expanded in Brazil.

In 2015, the Lancet Commission on Global Surgery reported that five billion people lack essential surgical care. The burden is even higher among low- and middle-income countries (LMICs), where nine out of ten people cannot access surgical services, and only 6% of surgeries occur worldwide. Investing in surgical care has also proved cost-effective since failure to increase funding for surgical scale-up can result in LMICs losing US$12.3 trillion by 2030^1^. This is pertinent for Brazil, a vast middle-income country that made strides to provide health services universally. Even though the Unified Health System is a role model for public health experts, it persists with substantial health disparities across socioeconomic segments^2^. In 2023, the GINI index for Brazil, an indicator of income inequality, stood at 53.4, positioning the country among the world’s most unequal societies^2^. It becomes evident that addressing national disparities requires a concerted effort to place surgical care strengthening to promote health and socioeconomic equality.

Despite Global Surgery being relatively novel, several initiatives were created to provide educational opportunities and disseminate knowledge on the field^3^. Although Brazilian researchers and institutions have contributed to the field’s expansion since 2015, Global Surgery education initiatives are still incipient in our country^4^. Hence, relying on successful strategies can be a starting point to promote the field among national surgical practitioners.

The first strategy relies on establishing formal training opportunities in Global Surgery^5^. In the United States, over 25 residency programs offer international clinical rotations, education, and research opportunities in Global Surgery^6^. Such an initiative demonstrates an increasing interest of trainees in seeking experiences within Academic Global Surgery. Other academic experiences consist of masters and fellowship programs^5^. One example is the fellowship from the Program in Global Surgery and Social Change, affiliated with Harvard Medical School, whose Brazilian laboratory has been committed to educating, researching, and mentoring over 100 Brazilian students in Global Surgery. Importantly, such training experiences must be skill-oriented. Recently, a consensus-based set of competencies was introduced to facilitate this process. These competencies serve as a guiding framework for educational programs in Academic Global Surgery and perioperative care to promote new experts in the field^7^. 

Additionally, enhancing international and national collaborations can increase research production and medical professionals’ interest in Global Surgery. HIC can provide academic program opportunities for healthcare professionals, and LMIC rotations can supply more contextually relevant learning than rotations in high-resource environments^5^. Many academic partnerships are role models in working to increase the number of surgeons in LMICs, such as the relationship between the College of Surgeons in East, Central, and Southern Africa (COSECSA) with the Royal College of Surgeons in Ireland (RCSI) and the American College of Surgeons (ACS). Creating an organized support team can escalate systems beyond a unique institution, and having financial resources can overcome time and geographical barriers^8^. 

As awareness and importance of Global Surgery increases in Brazil, there is a need to implement educational programs and initiatives for both aspiring and current surgeons. The Brazilian College of Surgeons has opened the door for this conversation in its latest congress, but this must lead to action. We propose a series of key recommendations aimed at fostering Global Surgery education at the national level:

1. Integrate Global Surgery into medical education and surgical residency: stimulate the creation of Global Surgery university programs or departments and include the subject as a formal curriculum component.
2. Structure the accreditation on Global Surgery education opportunities: define the competencies and skills to be developed and standardize national training quality.
3. Promote national research in Global Surgery: promote academic value, financial backing, and protected research time. The creation of Global Surgery-oriented fellowships, masters and doctoral programs should also be considered.
4. Strengthen capacity-building efforts: continuously encourage medical societies to actively incorporate surgical training courses into their conferences, promote seminars and educational events, and foster skill development and knowledge dissemination to implement Global Surgery actions among specialties.
5. Stimulate academic partnerships with HICs and LMICs: long-lasting collaborations between academic institutions promote innovation that builds capacity, strengthens health systems, and expands access to safe surgical treatment.

The expansion of Global Surgery lays the foundations to leverage and promote education and training opportunities, which will benefit surgical patients in the long term. The latest Brazilian Surgical Congress provided a space to rethink how solidifying Global Surgery into academic and surgical training can be an excellent addition for surgical practitioners, and several pathways exist to cultivate a new generation of Global Surgery leaders in the country.
